# Development of Methods to Evaluate Several Levels of Uranium Concentrations in Drainage Water Using Total Reflection X-Ray Fluorescence Technique

**DOI:** 10.3389/fchem.2019.00152

**Published:** 2019-03-22

**Authors:** Tsugufumi Matsuyama, Yukie Izumoto, Kota Ishii, Yasuhiro Sakai, Hiroshi Yoshii

**Affiliations:** ^1^Department of Radiation Measurement and Dose Assessment, National Institute of Radiological Science, National Institutes for Quantum and Radiological Science and Technology, Chiba, Japan; ^2^Department of Physics, Faculty of Science, Toho University, Chiba, Japan

**Keywords:** uranium, TXRF, drainage water, easy evaporator, Fukushima Daiichi nuclear power plant accident

## Abstract

As a country's law stipulates the effluent standard uranium concentration in drainage water, the uranium concentration must be determined when drainage water is released from a uranium handling facility, such as the Fukushima Daiichi nuclear power plant. The maximum allowable limit for uranium release at each facility is defined taking into consideration the situation of the facility, such as 1/10 to 1/100 of this effluent standard value. Currently, the uranium concentration of drainage water is commonly determined by α-particle spectrometry, in which several liters of drainage water must be evaporated, requiring about half of a day followed by 2–3 h of measurements, due to the low specific radioactivity of uranium. This work proposes a new methodology for the rapid and simple measurement of several levels of uranium in drainage water by a total reflection X-ray fluorescence (TXRF) analysis. Using a portable device for TXRF measurements was found to enable measurements with 1/10 the sensitivity of the effluent standard value by 10 times condensation of the uranium-containing sample solution; a benchtop device is useful to measure uranium concentrations <1/100 of the effluent standard value. Therefore, the selective usage of methods by a portable and benchtop devices allows for screening and precise evaluation of uranium concentrations in drainage water.

## Introduction

On March 11, 2011, an earthquake of magnitude 9.0 and subsequent tsunami caused a severe accident at the Fukushima Daiichi nuclear power plant, which then triggered a nuclear meltdown. The damaged reactor core was continuously cooled by instilling water; the water was contaminated with radioactive materials (e.g., actinides, fission products). During decommissioning, the contaminated water at the Fukushima Daiichi nuclear power plant will be drained after the concentration of each radioactive material is measured. The concentrations of radioactive materials in solutions are generally determined by measuring the emitted radiation, i.e., the α particles, β particles, and γ rays. This process allows for rapid and convenient measuring of radioactive materials with short half-lives; however, most uranium isotopes (i.e., ^238^U, ^235^U, and ^234^U) have long half-lives and rarely emit these radiations. Thus, a large volume of drainage water is normally condensed and the emitted radiation is then measured, requiring about half of a day and several hours, respectively.

The relationship between the number of atoms (*N*) and the radioactivity (*A*) is

(1) $$\begin{array}{r} N=\frac{T_{1/2}}{\text{ln}2}\;A, \end{array}$$

where *T*_1/2_ is the half-life of the radionuclide. According to Equation (1), the number of atoms per unit radioactivity for a radionuclide with a longer half-life is greater than that with a shorter half-life. Therefore, analytical methods whose signal intensity depends on *N* (e.g., X-ray fluorescence (XRF) analysis, inductively coupled plasma mass spectrometry, and inductively coupled plasma optical emission spectrometry) can more effectively measure uranium than those depending on the number of emitted radiation. To analyze trace elements, inductively coupled plasma mass spectrometry and inductively coupled plasma optical emission spectrometry are usually used; however, these require an onerous pretreatment to remove organic substances (El Himri et al., 2000; Unsworth et al., 2001; Daneshvar et al., 2009; Krachler and Carbol, 2011). A new uranium measurement method was thus developed using total reflection X-ray fluorescence (TXRF) analysis (Matsuyama et al., 2017, 2018; Yoshii et al., 2018). XRF measurements quantitatively and qualitatively analyze a trace element by measuring the secondary X-rays emitted after irradiating a sample with X-rays. TXRF analysis is an advanced technique of the XRF analysis using the total reflection phenomenon of X-ray on the surface of the materials.

Compton (1923) discovered X-ray total reflection. When an electromagnetic wave travels through an object, the refractive index can be explained as:

(2) $$\begin{array}{l} n=1+\frac{Nq_{e}^{2}}{2\epsilon_{0}m\left(\omega_{0}^{2}-\omega^{2}\right)}, \end{array}$$

where *N* is the number of atoms per unit volume of the object, *q*_*e*_ is the charge of an electron, ϵ_0_ is the permittivity, ω_0_ is the resonant frequency of an electron bound in an atom, and ω is the frequency of the electromagnetic wave (Feynman et al., 1963). In this case, the absorption of the X-ray into the object is not considered. As the frequency of an X-ray is much higher than that of the electron bound (i.e., ω ≫ ω_0_), $\omega_{0}^{2}$ can be ignored and (ω${}_{0}^{2}$-ω^2^) becomes negative. Replacing the absolute value of the second term with δ, the refractive index for the X-ray, *n*_*X*_, can then be given as Ais-Nielsen and McMorrow (2011):

(3) $$\begin{array}{l} n_{X}=1-\delta . \end{array}$$

As previously indicated (Klockenkämper et al., 1992), the order of δ is 10^−6^; *n*_*X*_ is thus slightly <1. When the X-ray enters the object from air at a glancing angle (90°-incident angle) that is less than the critical angle, θ_*c*_, the total reflection condition of the X-ray is satisfied. Using Snell's law, θ_*c*_ can be expressed as Equation (4) below and is on the order of 1 mrad (Wang et al., 2003).

(4) $$\begin{array}{l} \theta_{c}\approx \sqrt{2\delta } \end{array}$$

For example, θ_*c*_ is 1.75 mrad (0.1°) for an X-ray of 17.5 keV on silicon (Wobrauschek, 2007). Using the total reflection phenomenon, Yoneda and Horiuchi developed a highly sensitive analytical TXRF measurement method for trace elements in liquid samples (Yoneda and Horiuchi, 1971); TXRF analysis had explained in detail in various reports and books [see (Klockenkämper and von Bohlen, 2015)]. When a small amount of sample solution was dropped on a smooth substrate and dried, primary X-rays with glancing angles smaller than the critical angle are completely reflected from the surface of the thin sample residue. For such a case, the background (BG) signal, which depends on the scattered X-ray, is much lower than that in conventional XRF analyses. Accordingly, this method has been applied to various liquid samples, including seawater (Misra et al., 2006), wine (Galani-Nikolakaki et al., 2002; Anjos et al., 2003), blood (Khuder et al., 2007), and leaching solutions from plastic toys (Kunimura and Kawai, 2010a) with a minimum detection limit (MDL) ranging from several parts per billion (ppb; ng/g) to several parts per million (ppm; μg/g).

Recently, an MDL of uranium of approximately 0.30 ppm was achieved with a portable TXRF spectrometer with a measurement time of 3 min (Matsuyama et al., 2017). When a sample solution is assumed to contain only ^238^U with a half-life of 4.468 × 10^9^ y, the corresponding radioactivity concentration of uranium was calculated as approximately 3.7 mBq/cm^3^, which is significantly lower than the effluent standard value (i.e., 20 mBq/cm^3^) outlined in the Act on Prevention of Radiation Hazards due to Radioisotopes of Japan (Ministry of Education Culture Sports Science and Technology Japan, 2000). When the sample solution also contains ^235^U and ^234^U, which have half-lives of 7.04 × 10^8^ y and 2.46 × 10^5^ y, respectively, the radioactivity concentration corresponding to the MDL of uranium is higher than 3.7 mBq/cm^3^, as the radioactivity per unit mass of ^235^U and ^234^U are larger than that of ^238^U. In light-water reactors, such as the Fukushima Daiichi nuclear power plant, the abundance ratio of ^235^U is usually lower than 5%. When the abundance ratio of ^235^U is 5%, that of ^234^U is 0.037% (International Atomic Energy Agency, 2002). Using Equation (1), the radioactivity concentration of uranium corresponding to 0.30 ppm can then be calculated as 30 mBq/cm^3^, which is larger than the effluent standard value for uranium. As the MDL is inversely proportional to the square root of the measurement time, an increased measurement time provides a decreased MDL (Matsuyama et al., 2018). Extending the measurement time to 7 min provides an expected MDL of 0.196 ppm; the corresponding radioactivity concentration is 19.6 mBq/cm^3^, which is lower than the effluent standard value for uranium in drainage water.

However, drainage water inspection at a part of uranium handling facilities is performed using a method with an MDL of 1/10 of the effluent standard value defined by Japanese law; this would require a measurement time extension of more than 11 h. Therefore, in the present study, a method involving sample condensation followed by TXRF measurement is proposed. In this method, a sample solution is condensed by dissolving the dried residue obtained with a simple evaporator in a smaller volume of solution than the original liquid volume. The relationship between the condensation ratio and the MDL is first obtained to determine the condensation ratio required to achieve the target value. The proposed method is then compared with a method proposed by Kuniumura and Kunimura and Kawai (2007), which repeatedly drops and heats a sample to obtain a low MDL. Then, a calibration plot is obtained for the determination of uranium concentration in contaminated water.

Some facilities require a method with an MDL of 1/100 of the effluent standard value. In these facilities, very long condensation or measurement times may still be required to achieve the targeted MDL. The use of a high-performance benchtop TXRF device might be useful to achieve these target values, although it must be installed in an analysis room, thus losing the ability to perform the measurements anywhere. To obtain the MDL of uranium, the benchtop device was used for TXRF measurements of solutions with various uranium concentrations. The findings are then compared and the choice of method is discussed according to the required level of uranium contamination.

## Experimental

### Portable TXRF Instrument

A portable total reflection X-ray spectrometer (200TX; Ourstex Co., Ltd.; Neyagawa, Osaka, Japan), whose basic characteristics have been previously reported (Kunimura and Kawai, 2010b; Kunimura and Amagasu, 2015), was used to rapidly measure the uranium in uranium-contaminated water. The maximum tube voltage and current of the X-ray tube with a tungsten anode is 40 kV and 0.2 mA, respectively. A silicon drift detector with an active area of 7 mm^2^ was used to measure the TXRF spectra. An analyte on a sample holder was irradiated with incident X-rays collimated by a waveguide placed between the sample holder and the X-ray tube. The size of the collimated incident X-ray beam was approximately 10 μm long and 10 mm wide. The glancing angle of the incident X-rays was set to 0.05°, which is smaller than the critical angle of the sample holder. Because a portable TXRF instrument can be used wherever there is a power source to connect to, it can be used outdoors as well. In addition, for use of the instrument, no energy calibration is required after transport to other measurements areas. A tube current and voltage of 0.2 mA and 40 kV, respectively, and an XRF measurement time of 180 s were used for each measurement performed.

### Benchtop TXRF Instrument

The benchtop TXRF device NANOHUNTER-II (Rigaku Co., Tokyo, Japan), which has a higher sensitivity than 200TX for uranium measurement, was used to measure the uranium concentration of the sample solutions. A high-power water-cooled X-ray tube with a molybdenum anode and silicon drift detector was used as the X-ray source and detector, respectively. The X-rays emitted from the X-ray tube were monochromatized using a multilayer mirror. Monochromatic X-rays have two components: the dominant Mo Kα line (17.48 keV) and higher-energy X-rays (near 30 keV). Since the energy of a Mo Kα line is slightly higher than the U L_3_ threshold (17.17 keV), this device is useful for observing the U Lα line. The glancing angle of the monochromatic X-rays was set to 0.05°. The X-ray tube with a molybdenum target was operated with a tube voltage and current of 50 kV and 12 mA, respectively. The counting time for obtaining the TXRF spectrum was 300 sec.

### Sample Preparation for Evaluating the Stability of the Condensation Ratio

A multielement standard solution (XSTC-1407) containing 10 ppm of uranium, copper, cobalt, cesium, and thorium from SPEX CertiPrep, Inc. (NJ, USA) was diluted with ultrapure water to obtain solutions with uranium concentrations of 0.0125, 0.025, 0.05, 0.1, and 0.2 ppm. For each solution, a 1,600, 800, 400, 200, and 100 μL sample was completely dried by an evaporator (Smart Evaporator; Biochromato, Inc.; Fuzisawa, Japan). The dried residue was then dissolved in 20 μL of a 10 ppm yttrium solution prepared by diluting a 1,000 ppm yttrium standard solution purchased from FUJIFILM Wako Pure Chemical Corporation (Osaka, Japan). Yttrium was used as an internal standard. The final uranium concentration in each diluted solution of dried residue was 1 ppm at different condensation ratios. The sample preparation conditions are listed in Table 1 with the required drying times. Four solutions were prepared for each uranium concentration (*n* = 4). A solution containing 1 ppm uranium and 10 ppm yttrium was also prepared without solution condensation; the results of the TXRF measurements for this sample were then compared with those for the condensed samples. An aliquot of the sample solution (10 μL) was dropped onto fluorine-coated quartz glass (Sigma Koki Co., Ltd.; Tokyo, Japan) and dried for 5–10 min.

**Table 1:** Preparation of sample at different condensation ratios with the same uranium concentration.

**Uranium concentration (ppm)**	**Volume of dried solution (μL)**	**Volume of dissolving solution (μL)**	**Condensation ratio**	**Uranium concentration after condensation (ppm)**	**Required time for drying solution (min)**
0.2	100	20	5	1	10
0.1	200	20	10	1	15
0.05	400	20	20	1	30
0.025	800	20	40	1	50
0.0125	1,600	20	80	1	100

### Sample Preparation for Comparison With Kunimura's Methods

To perform a highly sensitive analysis of a trace element, Kunimura and Kawai (2007) compared a method in which a sample solution was repeatedly dropped and dried (M1) with a method in which the total amount of sample solution was dropped and dried (M2) using bottled water as the sample solution. To compare these methods with the proposed method, a sample solution containing 0.1 ppm uranium and 1 ppm yttrium was prepared. An aliquot of the sample solution (10 μL) was dropped onto a fluorine-coated optical flat and dried. This process was repeated 10 times for method M1 (*n* = 4). For method M2, a 10 times larger amount of the sample solution (100 μL) was dropped onto a fluorine-coated optical flat and dried (*n* = 4). As described in section Sample Preparation for Evaluating the Stability of the Condensation Ratio, a uranium solution with a 10 times higher concentration was prepared by drying 200 μL of a 0.1 ppm uranium solution and diluting it with 20 μL of a 10 ppm yttrium solution. By dropping 10 μL of this solution onto the fluorine-coated optical flat and drying it, three types of samples for the TXRF measurements with the same amounts of uranium (10 ng) and yttrium (100 ng) were prepared.

### Sample Preparation for Obtaining the Calibration Plots

Uranium solutions with concentrations of 0, 0.0625, 0.125, 0.25, 0.5, and 1 ppm were obtained by diluting XSTC-1407 with ultrapure water. The Smart Evaporator was used to dry 600 μL of the uranium-contaminated water; the dried residue was then dissolved in 60 μL of a diluted yttrium solution (10 ppm). Thus, the condensation ratio was 10. The samples for the TXRF measurements by the 200TX were prepared by dropping 10 μL of the uranium-contaminated water onto fluorine-coated quartz optical flats and drying them for 5–10 min (*n* = 4).

XSTC-1407 was diluted with ultrapure water to 0, 0.01, 0.05, 0.1, and 0.2 ppm of uranium concentration for high-sensitivity analysis using NANOHUNTER-II. Yttrium is not appropriate for usage as an internal standard here because the BG intensity around the energy region of the Y Kα peak is high in the spectrum obtained by NANOHUNTER-II. Therefore, a gallium standard solution purchased from FUJIFILM Wako Pure Chemical Corporation (Osaka, Japan) was used as the internal standard. Before the TXRF measurement, 190 μL of uranium solutions were mixed with 10 μL of 100 ppm gallium standard for a final gallium concentration of 5 ppm. A small portion (10 μL) of the mixture was dropped onto a fluorine-coated glass slide (Matsunami Glass Ind., Ltd., Osaka, Japan) and dried for about 5 min. The dried samples were measured by NANOHUNTER-II at a tube voltage, tube current, and XRF counting time of 50 kV, 12 mA, and 5 min, respectively.

## Results and Discussion

### Evaluation of Condensation Ratio

**Figure 1:**
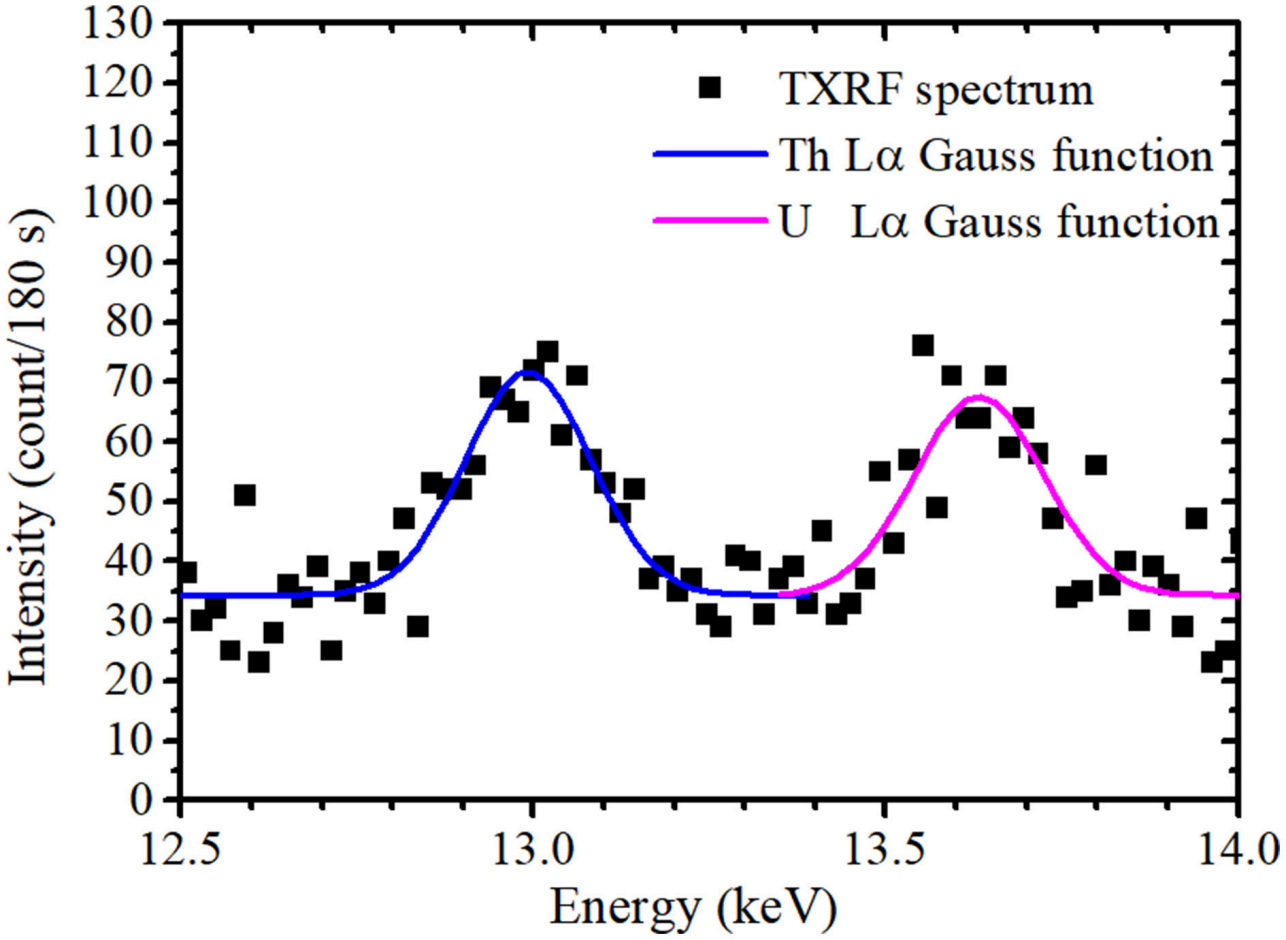
Magnified TXRF spectrum with a condensation ratio of 10 around and the U Lα and Th Lα peaks reproduced by Gaussian function.

**Figure 2:**
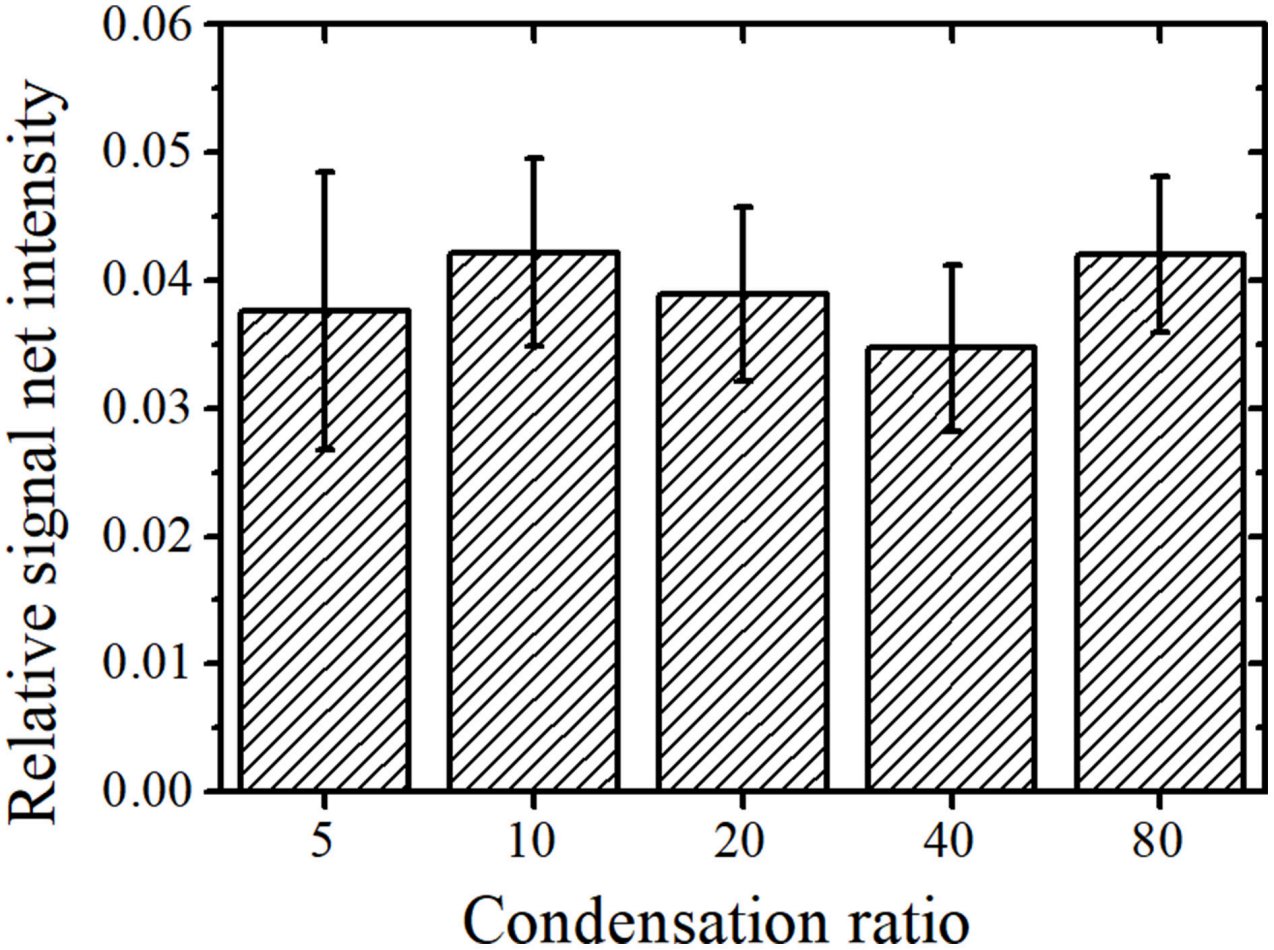
Condensation ratio vs. the average relative net intensity with standard deviation (SD).

A typical TXRF spectrum of the 10 times-condensed sample measured by portable TXRF device at an energy range between 12.5 and 14.0 keV is shown in Figure 1. Peak fitting using the Gaussian function was performed to obtain the net intensity of the U Lα peak, as XRF element peaks can generally be described using a first-order approximation by the Gaussian function. Since the XRF peak contains BG in the TXRF spectrum, the function of an XRF peak is determined as

(5) $$\begin{array}{l} f_{gross}=y_{0}+\frac{A}{w\sqrt{\frac{\pi }{2}}}\exp\left(-\frac{2{\left(x-x_{c}\right)}^{2}}{w^{2}}\right), \end{array}$$

where *x* indicates the X-ray energy, *y*_0_ is the BG intensity, *A* is the height parameter, *w* is the peak width, and *x*_*c*_ is the reference value of the characteristic X-ray. The relationship of *w* and the full width at half maximum (FWHM) of the peak is given by *w* = $\frac{FWHM}{\sqrt{2ln2}}.$ As shown in Figure 1, there are two peaks at the Th Lα and U Lα lines at 12.97 and 13.61 keV, respectively. An equivalent *y*_0_ was used for function determination of the Th Lα and U Lα peaks, as they had a similar BG intensity. As previously reported (Matsuyama et al., 2018), Gaussian fitting using only *y*_0_, *A*_Th_, and *A*_U_ as fitting parameters with fixed *w*_Th_ and *w*_U_ values obtained by long-time measurements converges easily. The *w* values of the Th Lα and U Lα peaks were assumed as 0.179 and 0.184 keV, respectively (Matsuyama et al., 2018). Integrating the Gaussian curve (fitting also shown in Figure 1) gives the net intensity of the U Lα peak. The BG intensity at the energy region of the U Lα peak can be calculated from *y*_0_ value. Additionally, Gaussian fitting was performed for the Y Kα peak (14.96 keV) to obtain its net intensity as an area of Gaussian function. The peak width was not fixed during fitting because the Y Kα peak was isolated with a high enough intensity to perform Gaussian fitting with *y*_0_, *A*_Y_, and *w*_Y_. Figure 2 shows the average relative signal net intensity (the net intensity of the U Lα peak divided by the net intensity of the Y Kα peak) and standard deviation (SD) for each condensation ratio. The relative net intensities were all similar within the range of error regardless of the condensation ratio; therefore, the sample solution could be successfully condensed in the entire region used in this study.

The MDL of an element can be calculated as follows:

(6) $$\begin{array}{l} \text{MDL}=\frac{3C}{I_{net}}\sqrt{\frac{I_{BG}}{t}}, \end{array}$$

where *C* is the concentration of an element (ppm), *I*_*net*_ is the net XRF intensity (counts per second, cps), *I*_*BG*_ is the BG intensity in the energy region of the corresponding XRF peak (cps), and *t* is the XRF counting time (s) (Kunimura and Ohmori, 2012; Kunimura et al., 2017).

**Figure 3:**
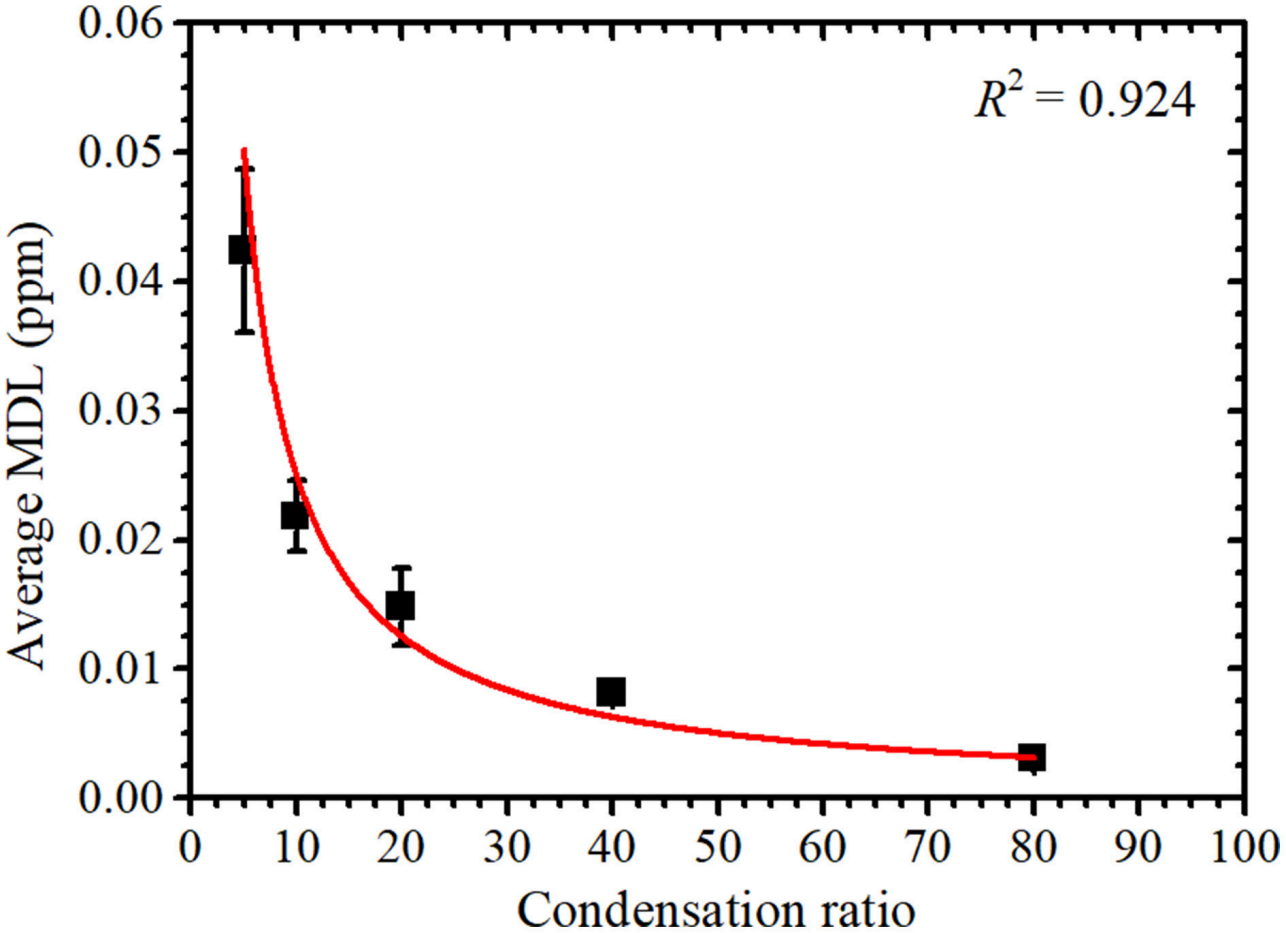
Condensation ratio vs. the average minimum detection limit (MDL) with a fitted curve to highlight the inversely proportional relationship.

As shown in Figure 3, the average MDL decreased with an increase in condensation ratio. As the final concentration of uranium in the condensed sample solutions were the same, the average value of *I*_*net*_ was similar for each sample solution. The average value of *I*_*BG*_ was also similar, and *t* was constant for each measurement. In this case, the value of *C* can be given as the uranium concentration before condensation and is thus inversely proportional to the condensation ratio. Therefore, the MDL is inversely proportional to the condensation ratio as

(7) $$\begin{array}{l} \text{MDL}=\frac{a}{\text{condensation ratio}}, \end{array}$$

where *a* is a proportionality constant, which is the MDL value for a 1 ppm uranium solution without condensation. From the fitting results obtained using Equation (7), also shown in Figure 3, *a* = 0.25 ± 0.02 ppm; the MDL of the 1 ppm uranium solution without condensation was thus 0.22 ± 0.05 ppm, and they are same within the range of error. An MDL of 0.3 ppm without condensation was previously reported (Matsuyama et al., 2017). As the optical flats used in this work were coated with a fluorine resin, the intensity of the U Lα peak was increased, as discussed by Nagai et al. (2014). From Equation (6), the net intensity is inversely proportional to the MDL; thus, the increase in net intensity caused a lower MDL without condensation.

Although the MDL decreases as the condensation ratio increases, the increased condensation ratio resulted in an increased condensation time, as indicated in Table 1. Therefore, the condensation ratio should be smaller and within a range that meets the required MDL. From the considered maximum abundances of ^235^U and ^234^U, the radioactivity concentrations of uranium corresponding to the obtained MDL at condensation ratios of 5, 10, 20, 40, and 80 were calculated as 4.2, 2.2, 1.5, 0.81, and 0.31 mBq/cm^3^, respectively. When the sample was condensed 10 times in concentration, the MDL was slightly greater than one tenth of the effluent standard value defined by Japanese law with a required condensation time of only 15 min.

### Comparison With Kunimura's Methods

In Kunimura and Kawai (2007) comparison of highly sensitive analysis methods (M1 and M2, as described in section Sample Preparation for Comparison with Kunimura's Methods) of the elements in bottled water, they concluded that repeatedly dropping and drying a sample of the solution (M1) was better in sensitivity than dropping and drying the total amount of the solution (M2). In the proposed method, the sample is condensed before dropping.

**Figure 4:**
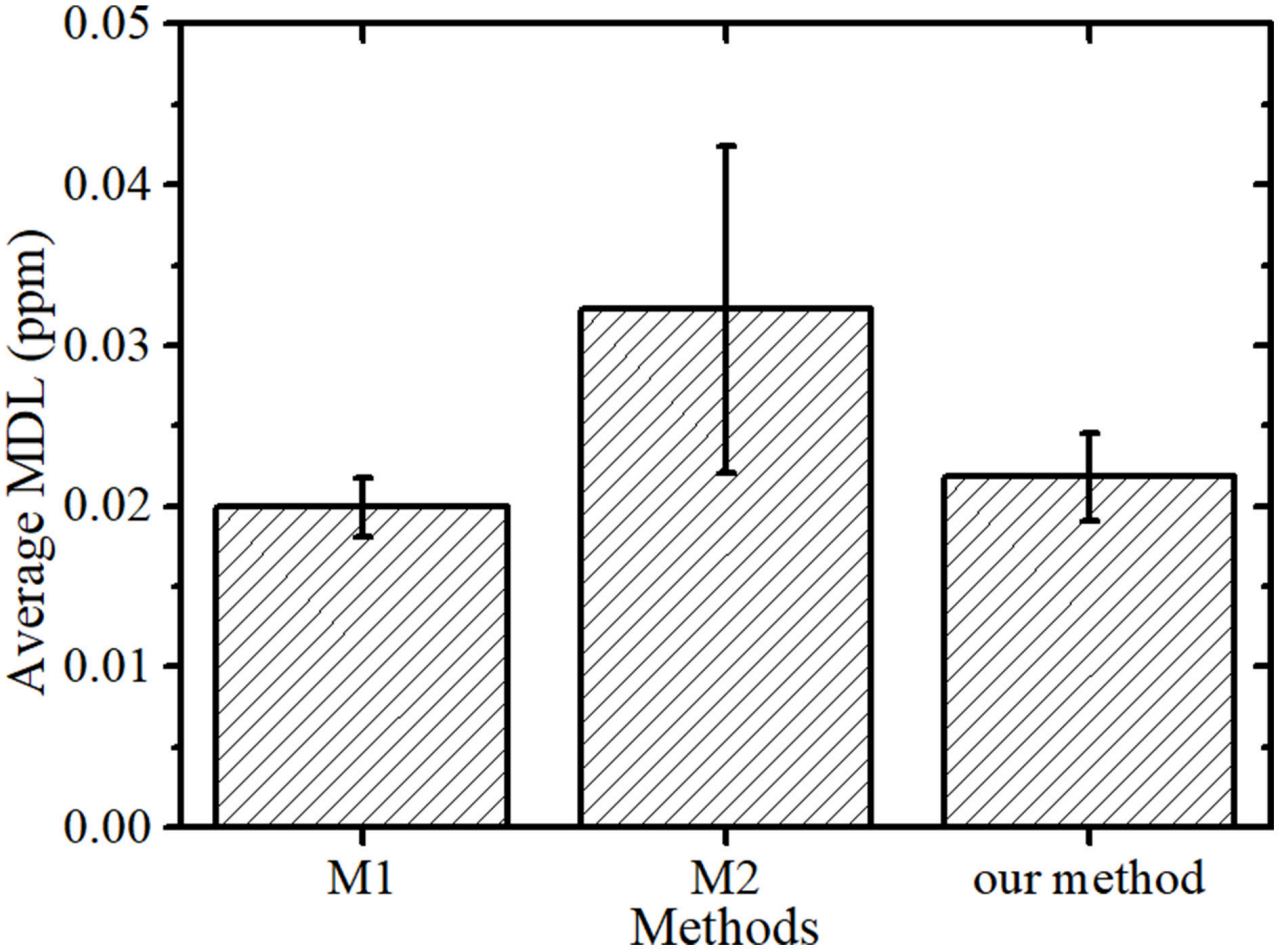
Average MDL and SD of the Kunimura's methods (M1 and M2) and the proposed method.

The average MDL and SD of M2 were greater than those of the other two methods, as shown in Figure 4. This was because the location of the dried residue varied greatly and the dried residue was approximately 12 mm in diameter, which was larger than the effective area of the detector. In contrast, the dried residues of the proposed method and M1 were 1–3 mm in diameter and the measurement loss of the XRF emitted from sample was lower than that of M2, resulting in similar resulting MDLs. However, the sample preparation times required for the proposed method and M1 were different. The repeated dropping and drying necessary for M1 required approximately 90 min, whereas the drying time using a simple evaporator and the time for dropping and drying the condensed sample on a substrate only needed approximately 15 min and 5–10 min, respectively. This shorter preparation time in the proposed method is advantageous for analyzing many samples.

### TXRF Spectra and Calibration Plot for the Condensed Uranium Solution

**Figure 5:**
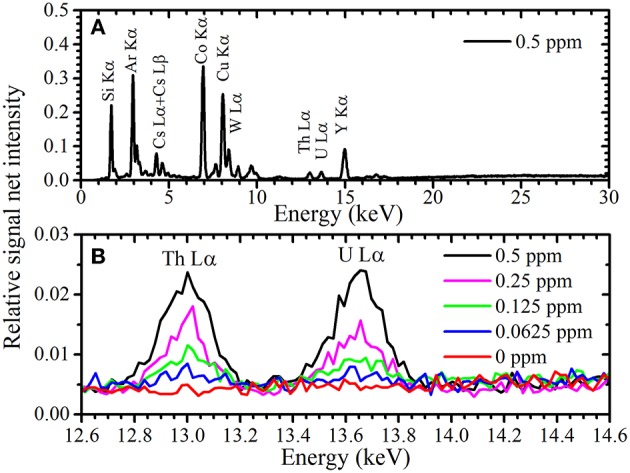
TXRF spectrum **(A)** of a sample solution containing 0.5 ppm uranium and **(B)** expanded around the U Lα peak.

The obtained TXRF energy spectrum of the uranium solution containing 0.5 ppm uranium condensed 10 times and normalized by the net intensity of the Y Kα peak is shown in Figure 5A. Figure 5B presents magnified views of the U Lα peak of the solutions containing 0, 0.0625, 0.125, 0.25, and 0.5 ppm uranium, each condensed 10 times. The Si Kα and Ar Kα peaks were observed due to the quartz glass substrate used as a sample holder and the presence of 0.9 vol% argon in air, respectively. The W Lα peak originated from a characteristic X-ray of X-ray tube scattered by the sample or substrate. Peaks corresponding to the uranium, thorium, cobalt, copper, and yttrium in the sample solution were also detected. The height of the U Lα peak shown in Figure 5B is seen to be proportional to the uranium concentration.

**Figure 6:**
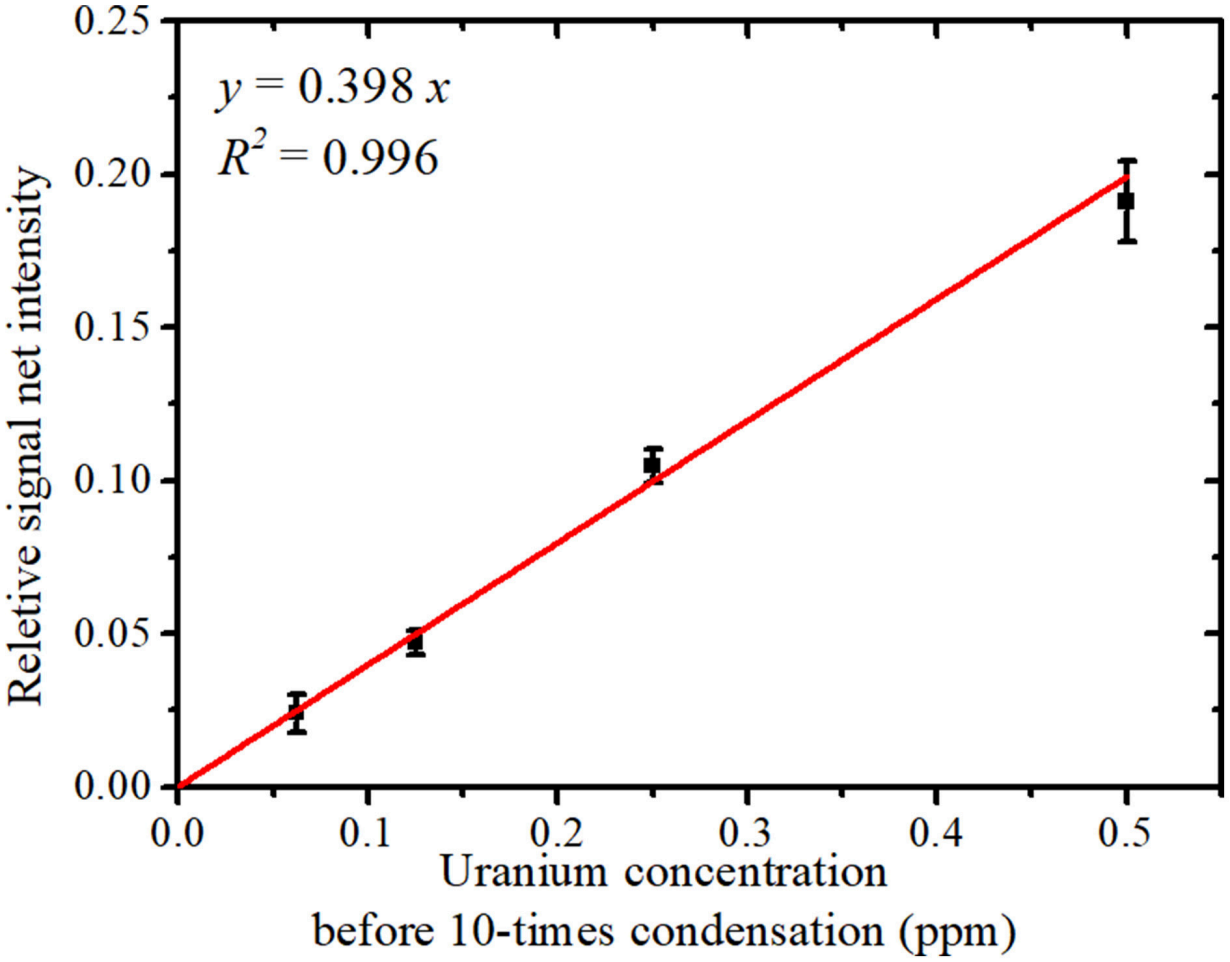
The relative signal net intensity (U Lα signal/Y Kα signal) vs. the solution uranium concentration.

The relationship between the uranium concentration in the sample solution before condensation and the average relative signal net intensity (U Lα net intensity/Y Kα net intensity) is shown in Figure 6; error bars represent the SD. The average relative signal net intensity was directly proportional to the uranium concentration over the entire concentration region. This calibration plot allows the uranium concentration of contaminated water to be determined from the relative net intensity.

### TXRF Spectra Measured by Benchtop Device

**Figure 7:**
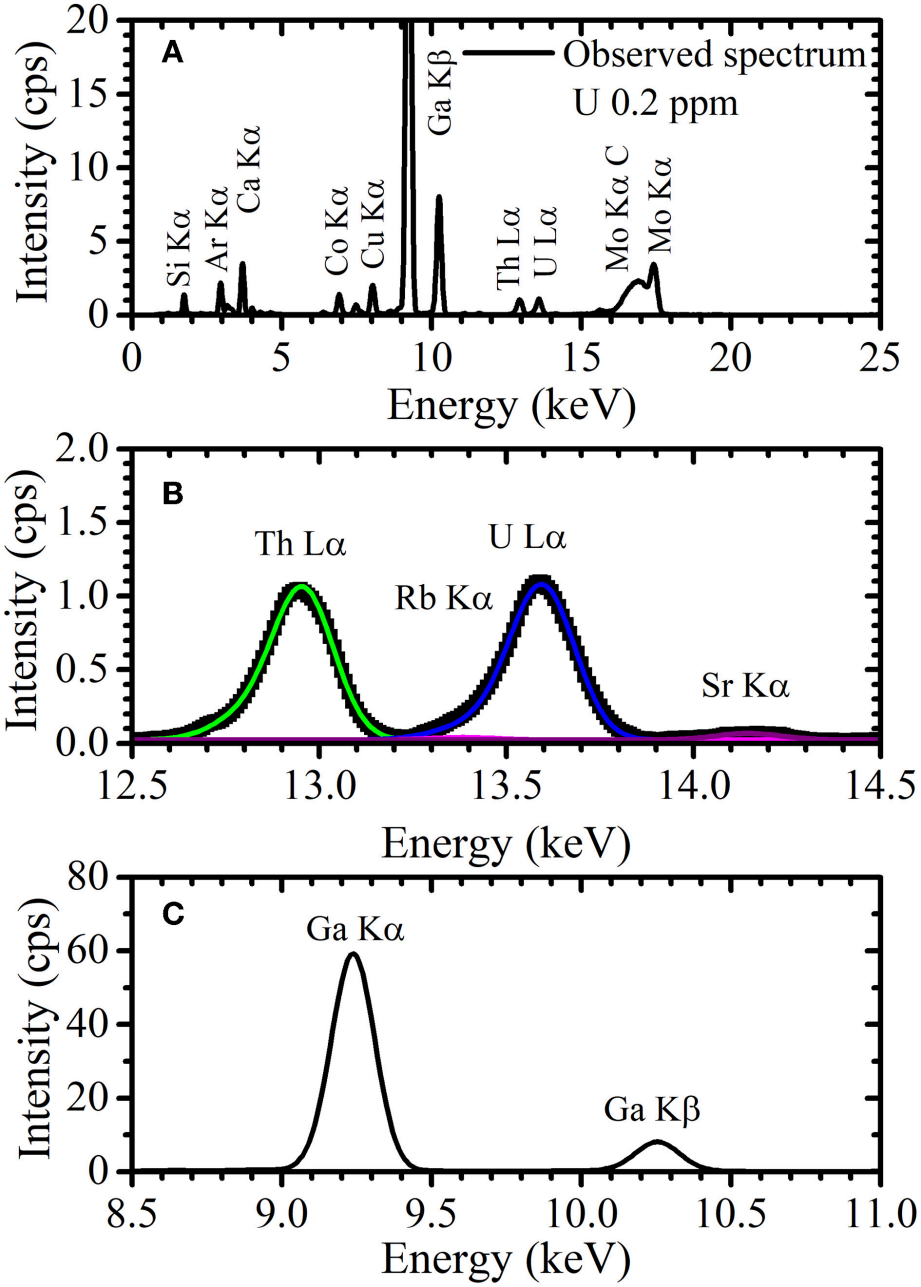
**(A)** The TXRF spectrum of a sample solution containing 0.2 ppm uranium and 5 ppm gallium and expanded views of the energy region around the **(B)** U Lα and **(C)** Ga Lα peak.

The TXRF energy spectrum of the solution containing 0.2 ppm uranium and 5 ppm gallium observed by NANOHUNTER-II is shown in Figure 7A. Similarly, Si Kα and Ar Kα peaks were observed. The Mo Kα and Compton scattering peak were detected, due to the scattering of the monochromatic X-ray from the molybdenum target X-ray tube by the sample or glass slide. An expanded view around the energy region of the U Lα and Ga Kα peaks is shown in Figures 7B,C, respectively. Glass slides sometimes contain an extremely small amount of rubidium and a small amount of strontium, although the quartz optical flats used for measurements with 200TX contained almost no rubidium or strontium. Thus, the Sr Kα peak was seen when using the benchtop device. Although an extremely low-intensity Rb Kα peak may also exist here (Figure 7B), it was not clearly detected because of overlap with the U Lα peak.

As shown in Figure 5, the Th Lα and U Lα peaks observed by the 200TX were symmetrical. Therefore, the Th Lα and U Lα peaks could be considered with a single Gaussian function and the peak widths were thus determined by simple Gaussian fitting. However, since the excitation efficiency of electrons in the U L_3_ and Th L_3_ shells were higher when observed with NANOHUNTER-II, the Th Lα and U Lα peaks each have two components: the Th Lα_1_ and Lα_2_ lines and the U Lα_1_ and Lα_2_ lines, respectively. Since the intensity of the Lα_2_ peaks were lower than those of the Lα_1_ peaks for Th and U, the Th Lα and U Lα peaks had shoulders originated by the Lα_2_ peaks. Additionally, the low-intensity Rb Kα peak was present in the energy region of the U Lα peak. To calculate the net intensities of the U Lα_1_ and Lα_2_ peaks, the components of these three peaks (i.e., the U Lα_1_, U Lα_2_, and Rb Kα) must be separated.

Peak widths of the Th Lα_1_, Th Lα_2_, U Lα_1_, and U Lα_2_ peaks in the TXRF spectrum observed by NANOHUNTER-II were determined by long-time measurement of 10 times diluted XSTC-1407 standard solution. The peak intensities for the Th Lα and U Lα lines in the diluted standard solution containing 1 ppm of thorium and uranium were much higher than that of the Rb Kα line in the rubidium-containing blank glass slide. Therefore, the Rb Kα peak was neglected in the double Gaussian fittings for these peaks using the Lα_1_ and Lα_2_ peaks for thorium and uranium, respectively. To determine the peak widths of the Rb Kα and Sr Kα lines, the TXRF measurements for the diluted solutions of rubidium standard solution and strontium standard solution (FUJIFILM Wako Pure Chemical Corporation, Osaka, Japan) were performed. Small portions (10 μL) of the diluted solutions were dropped onto glass slide, dried, and then measured by the NANOHUNTER-II. The tube voltage, tube current, and measurement time were 50 kV, 12 mA, and 3,600 s, respectively. From the Gaussian fittings, the widths of Th Lα_1_, Th Lα_2_, Rb Kα, U Lα_1_, U Lα_2_, and Sr Kα peaks were 0.164, 0.176, 0.174, 0.172, 0.212, and 0.180 keV, respectively. After fixing the peak width to these values, sextuple Gaussian fittings were performed for the TXRF spectra of the uranium solution as analyzed by NANOHUNTER-II. As shown in Figure 7B, overlapping peaks were separated by Gaussian fitting with fixed peak widths. The Gaussian fitting for the Ga Kα peak (9.25 keV) was also performed.

**Figure 8:**
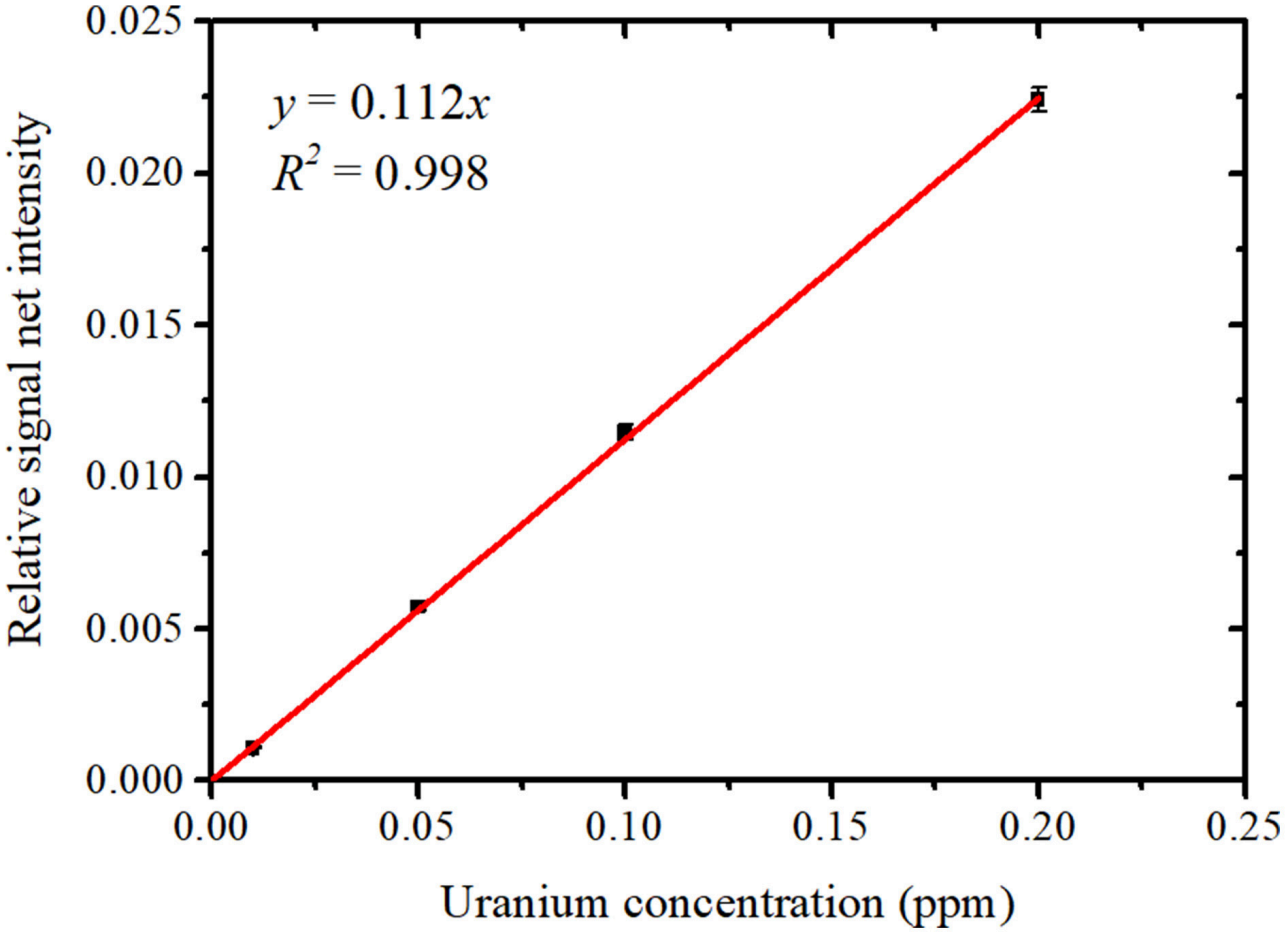
Developed calibration plot between the uranium concentration and relative signal net intensity (U Lα / Ga Lα).

The areas of the Gaussian peaks of the U Lα_1_ and U Lα_2_ lines were then added together to obtain the net intensity of the U Lα peak. Although the net intensity of the Rb Kα peak was also calculated as an area of the Gaussian function, it was much lower than the net intensity of the U Lα peak even for the sample with the lowest uranium concentration, 0.01 ppm. The net intensity of the Ga Kα peak was also obtained as an area of the Gaussian function. Figure 8 shows the relationship between the uranium concentration and the average relative net intensity, calculated as the net intensity of the U Lα peak normalized by that of the Ga Kα peak. The error bars represent the standard deviation of the four data points for each uranium concentration. The relative signal net intensity was found to be proportional to the uranium concentration within the region used. The MDL for uranium was calculated as 1.4 ppb. The radioactivity concentration for uranium corresponding to this MDL at the maximum abundances of ^235^U and ^234^U in a light-water reactor was thus 0.14 mBq/cm^3^, which is lower than 1/100 of the effluent standard value defined by Japanese law. Therefore, the method using NANOHINTER-II allows for highly sensitive determination of uranium concentration in a sample solution. In addition, this method can take highly sensitive measurements for radionuclides with long half-lives.

### Choice of Measurement Method According to the Required Uranium Detection Levels

Using Equation (6), MDL value for various measurement time can be estimated because MDL value is inversely proportional to the square root of the measurement time. The portable TXRF device used to measure uranium-contaminated water provided a detection limit estimated as lower than the effluent standard value when the measurement time is set to 7 min. With 10 times condensation, the obtained MDL of uranium in a 3 min measurement using the portable device was about 1/10 of the effluent standard value. To ensure a detection limit of uranium of lower than 1/10 of the effluent standard value, the measurement time for a 10 times condensed sample should be extended to 4 min. A detection limit of <1/10 of the effluent standard value was also given for a 3 min measurement of 20 times condensed sample. However, condensation time in the case of 10 times condensation is 15 min, whereas 20 times condensation requires 30 min. Thus, 4 min measurement of the 10 times condensed sample has advantage in analysis time. A major advantage of this method is its portability; these measurements can thus be performed anywhere. However, the maximum allowable limit for uranium release of some facilities handling various radionuclides is set at 1/100 or less of the effluent standard value. To achieve this level using portable device by increasing condensation ratio and extending the measurement time, a sample condensed 40 times measured for 50 min is fastest, although it would require over 100 min in total including prep time. Performing the same analysis *in situ* takes too long. Therefore, a benchtop device should be used, even if portability is lost. The MDL of uranium using the benchtop device with a 5 min measurement was about 1/140 of the effluent standard value.

An analytical method that can detect uranium in drainage water at a sensitivity of 1/100 of the effluent standard value is needed for the demolition of the Fukushima Daiichi nuclear power plant, thus requiring the usage of the benchtop device. However, sample solutions contaminated with high concentrations of uranium should not be brought into a clean analysis room. Therefore, the portable device offers the possibility to analyze a possibly uranium-contaminated liquid condensed 10 times *in situ*. If significant amounts of uranium are detected by this method, the solution can then be diluted there. This diluted solution can then be safely brought back to the analysis room along with collected samples not detected to have uranium with the portable device for precise analysis with the benchtop TXRF device.

## Conclusion

A new methodology for the rapid and simple measurement of several levels of uranium in drainage water by total reflection X-ray fluorescence (TXRF) analysis was proposed that uses a portable device for initial screening and a benchtop device for more precise measurement of uranium contamination. An MDL of uranium using a benchtop TXRF spectrometer of 1.4 ppb, corresponding to <1/100 of the effluent standard value stipulated by Japanese law, was obtained in the analysis time including sample drying and measuring of 10 min. Furthermore, condensation of the sample with a simple evaporator also allowed for the detection of uranium in drainage water with a sensitivity of 1/10 of the effluent standard value even in portable equipment. Total analysis time using the portable equipment, including condensation time, drying time, and measurement time, was estimated to be 25 min and can be performed *in situ*. Therefore, a simple measurement methodology was proposed to use either the portable or the benchtop device according to the required measurement sensitivity. In the decommissioning of the Fukushima Daiichi nuclear power plant, the portable, *in situ* TXRF device with sample condensation can determine whether dilution is necessary to safely bring the sample into the clean analysis room. The benchtop device can then be used to perform the more precise TXRF analysis. These methods can be used for drainage water management in various uranium handling facilities, including the decommissioning field of the Fukushima Daiichi nuclear power plant.

## Author Contributions

TM research planning, sample preparation for the TXRF measurements and data analysis, and manuscript preparation. YI assistance with the sample preparation, discussion, and manuscript review. KI assistance with the TXRF measurements. YS assistance with the planning, discussion, and manuscript review. HY research planning, data analysis, and manuscript review.

### Conflict of Interest Statement

The authors declare that the research was conducted in the absence of any commercial or financial relationships that could be construed as a potential conflict of interest.
